# Early First-Trimester Diagnosis of Thoracopagus Conjoined Twins with a Shared Cardiac Structure

**DOI:** 10.3390/diagnostics16162562

**Published:** 2026-08-14

**Authors:** Adelina Staicu, Camelia Albu, Roxana Constantin, Iulian Gabriel Goidescu, Mihai Surcel, Dan Boitor-Borza, Andreea Florian, Georgiana Irina Nemeti, Gheorghe Cruciat, Daniel Muresan, Ioana Cristina Rotar

**Affiliations:** 1 1st Department of Obstetrics and Gynecology, “Iuliu Haţieganu” University of Medicine and Pharmacy Cluj-Napoca, 400012 Cluj-Napoca, Romania; staicuadelina@gmail.com (A.S.);; 2 Centre of Advanced Research Studies, Emergency County Hospital “IMOGEN”, 400006 Cluj-Napoca, Romania

**Keywords:** thoracopagus conjoined twins, first-trimester ultrasound, prenatal diagnosis, shared heart, color Doppler

## Abstract

Thoracopagus conjoined twins are the most common type of conjoined twinning and are frequently associated with shared cardiac anatomy, the principal determinant of prognosis and postnatal survival. Early prenatal diagnosis is essential for accurate counseling and timely pregnancy management. We report the diagnosis of thoracopagus conjoined twins with a shared cardiac structure in a 35-year-old primigravid woman at 8 weeks and 3 days of gestation. High-resolution transvaginal ultrasound demonstrated persistent thoracic fusion, fixed relative fetal position, a single yolk sac, a single umbilical cord, and absence of an intertwin membrane. Color Doppler confirmed a single sonographically detectable cardiac complex, while three-dimensional ultrasound enhanced spatial visualization and facilitated parental counseling. Differential diagnoses, including monochorionic twins with close apposition, TRAP sequence, body stalk anomaly, and other forms of conjoined twinning, were systematically excluded. Early first-trimester recognition of thoracopagus conjoined twins with shared cardiac anatomy is feasible using dynamic ultrasound and Doppler imaging. Establishing the diagnosis before the end of the first trimester enables accurate prognostic assessment, timely multidisciplinary counseling, and informed parental decision-making for this condition associated with an extremely poor prognosis.

**Figure 1:**
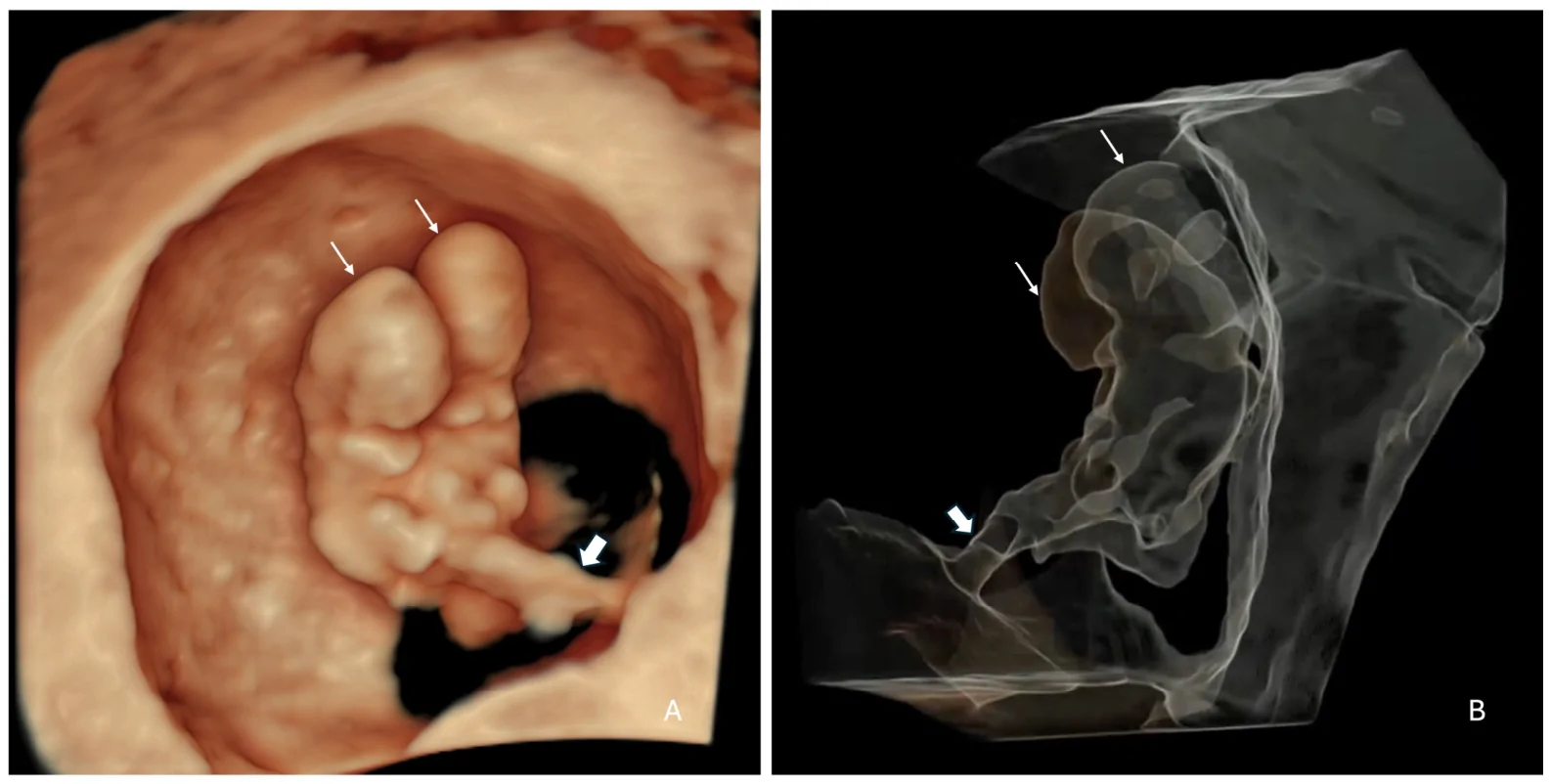
High-resolution transvaginal ultrasound using 3D and Silhouette reconstruction of a 35-year-old primigravida who presented for routine confirmation of a spontaneously conceived pregnancy. Three-dimensional transvaginal ultrasound was performed using a Voluson™ E10 ultrasound system (GE HealthCare, Zipf, Austria) equipped with a RIC5-9-D volumetric transvaginal transducer. Volume datasets were acquired using a 60° acquisition angle, a 62° B-mode sector angle, and a scanning depth of 4.6 cm, with a volume rate of 33 Hz. Safety indices were maintained at a Mechanical Index (MI) of 0.7, a Thermal Index for soft tissue (TIs) of 0.3, and a Thermal Index for bone (TIb) of 0.3. The acquired volumes were reconstructed using both HDlive (**A**) and Silhouette (**B**) rendering modes. HDlive rendering was performed using the obstetric preset (HDlive/OB), with Quality set to Max, Mix 70/30, CRI level 2, and V-SRI level 4. Silhouette rendering was performed with Quality set to Max and CRI level 2. First-trimester transvaginal ultrasound performed at 8 weeks and 3 days of gestation demonstrated two embryos with crown–rump lengths of 1.96 cm and 2.21 cm, respectively. The embryos remained in a constant position and exhibited two distinct cephalic poles (narrow arrows), a single ventrally fused body at the thoracic level, four superior upper limbs, and four inferior lower limbs (**A**). A single yolk sac, a single umbilical cord (thick white arrow), and a single placental mass without an intertwin membrane were identified (**B**), sustaining the diagnosis of thoracopagus conjoined twins (Supplementary Video S1).

**Figure 2:**
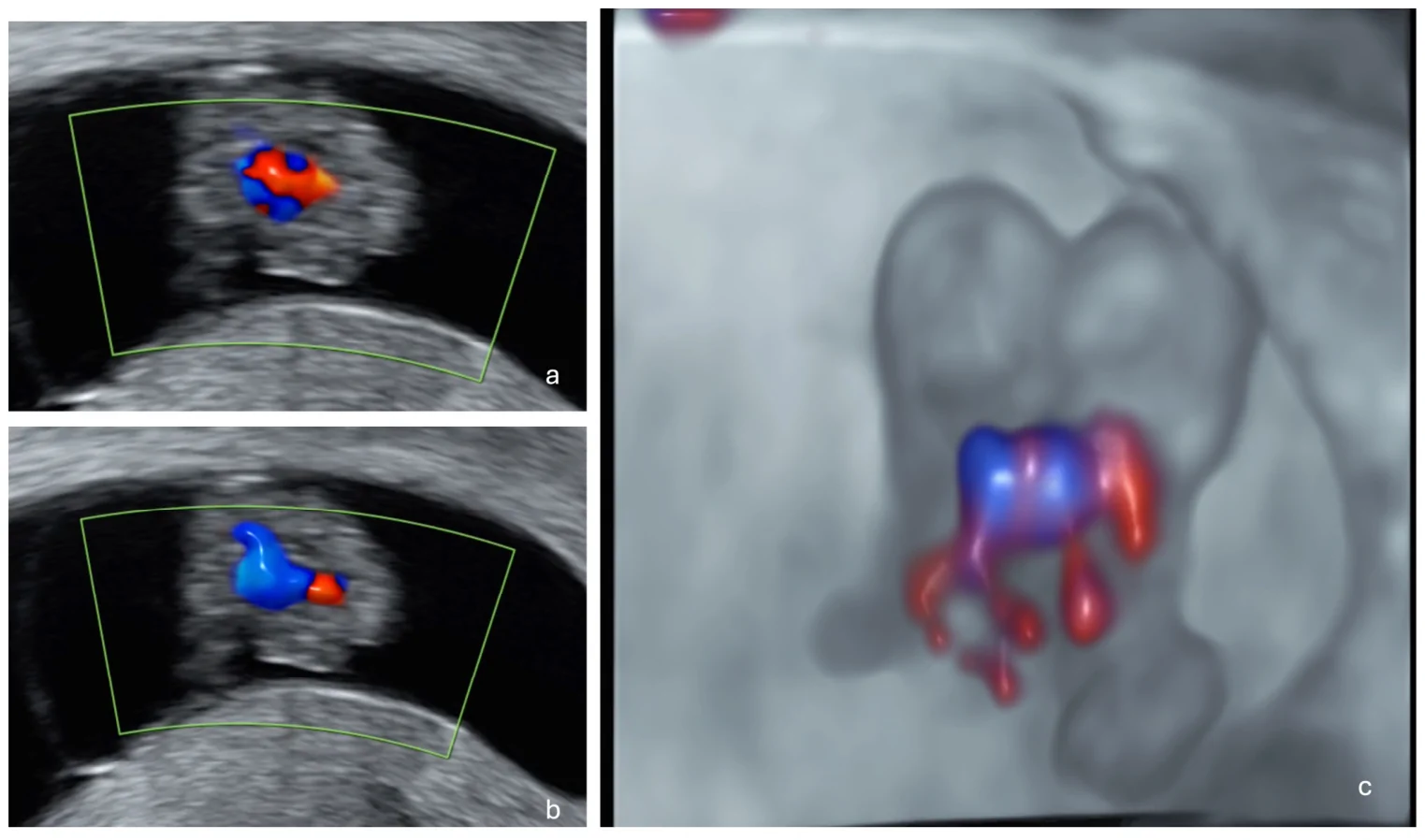
Cardiac assessment of 8-week and 3 days thoracopagus conjoined twins using a Voluson E10 ultrasound system (GE HealthCare, Zipf, Austria) equipped with an RM7C transvaginal volumetric transducer. (**a**,**b**) 2D color Doppler demonstrates a single sonographically detected cardiac structure located in the fused thoracic region. (**c**) Three-dimensional HD-Flow™ datasets acquired using the Glass Body rendering mode. Acquisition parameters included a B-mode angle of 18°, a volume angle of 40°, an imaging depth of 6.1 cm, and V-SRI level 3. During the examination, the Mechanical Index (MI) was 1.2, while the Thermal Index for soft tissue (TIs) and bone (TIb) were both 0.5, in accordance with routine obstetric imaging safety recommendations. Because of the early gestational age, the examination did not permit definitive anatomical characterization of the cardiac complex (Supplementary Video S2). To finalize the diagnosis and provide appropriate parental counseling, several differential diagnoses were considered and sequentially excluded. The main differential diagnoses at this gestational age include non-conjoined monochorionic monoamniotic twins, the twin reversed arterial perfusion (TRAP) sequence, body stalk anomaly, and other forms of conjoined twinning. At small crown–rump lengths, twin embryos may appear closely positioned with limited independent movement, potentially mimicking fusion [1]. In the present case, the diagnosis of thoracopagus conjoined twins was supported by two separate cephalic extremities, relatively fixed embryo positioning during real-time examination, and persistent ventral fusion of the embryos at the thoracic level, strengthened by the identification of a single umbilical cord, the absence of a separating membrane, and a single yolk sac [2]. In the present case, the TRAP sequence diagnosis was excluded based on the presence of two relatively symmetrical embryos with distinct cephalic poles, persistent thoracic fusion, and a single functioning cardiac structure within a common thoracic mass, together with the absence of the characteristic pump-twin/acardiac twin morphology and reverse arterial perfusion [3]. Other forms of conjoined twinning, such as dicephalus or craniopagus twins, may be considered in the differential diagnosis when two cephalic structures are identified [3]. Dicephalus twins are characterized by a single trunk with two heads, whereas craniopagus twins demonstrate fusion limited to the cranial region, with separation of thoracic and abdominal structures. In thoracopagus conjoined twins, fusion predominantly involves the thoracic region and is frequently associated with shared vital organs. Severe fetal malformation complexes, including body stalk anomaly or limb–body wall complex, may occasionally mimic aspects of conjoined twinning in early gestation [4]. These conditions are typically associated with marked disruption of normal fetal anatomy, an absent or extremely short umbilical cord, and a lack of organized bilateral fetal structures. In contrast, conjoined twins demonstrate recognizable embryonic organization, bilateral symmetry of cephalic structures, and a defined umbilical cord, as observed in this case. Following comprehensive evaluation and exclusion of the relevant differential diagnoses, the combined two-dimensional ultrasound, color Doppler, and three-dimensional findings established the diagnosis of thoracopagus conjoined twins with a shared cardiac complex. Following multidisciplinary, non-directive counseling regarding the extremely poor prognosis, the anticipated inability to achieve successful separation, and the available management options, the patient elected termination of pregnancy. Pregnancy termination was subsequently recommended and performed at 9 weeks of gestation using mifepristone and misoprostol.

**Figure 3:**
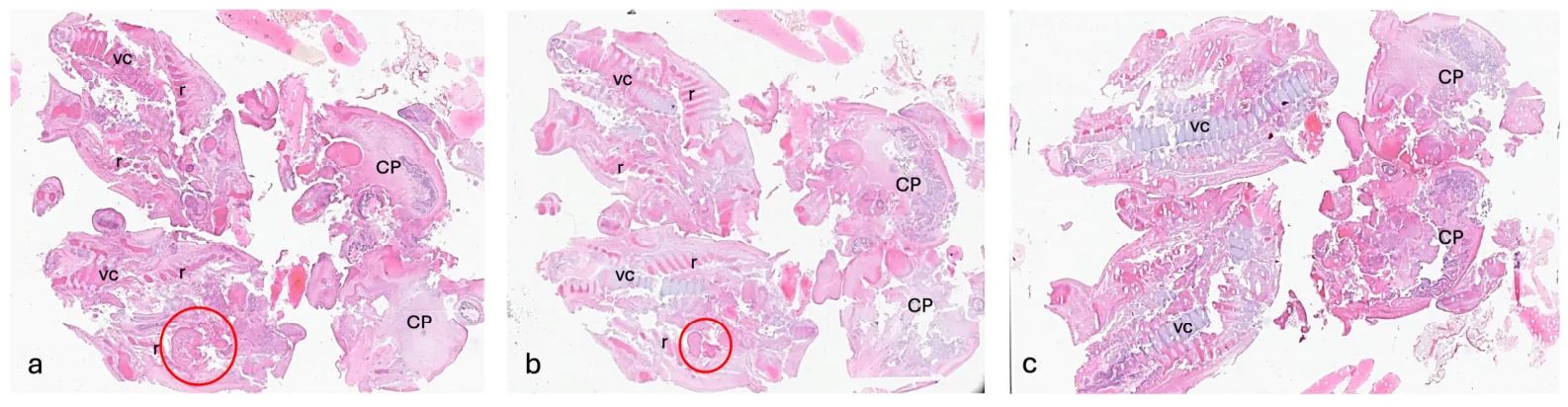
Due to the small gestational age and severe tissular friability, the fetuses arrived partially fragmented, with the two cephalic poles detached. The ventrally fused trunks at the thoracic level and the four limbs were mostly intact. The microscopic examination was performed on the two sections obtained after performing one coronal section of the trunk. The sections were processed using a vacuum infiltration processor, namely Tissue-Tek VIP 5 Jr (Sakura, Alphen an den Rijn, The Netherlands), and embedded in paraffin blocks. To obtain panoramic views, the slides were scanned using a slide scanner (Aperio GT 450 Dx, Leica Biosystems, Deer Park, IL, United States). The images were acquired using dedicated digital slide viewer software (Aperio Slide Manager Version 12.6.0.5007 UDI 00815477020402(8012)12.6, Leica Biosystems, Deer Park, IL, United States). HE (**a**,**b**) 0.4×; (**c**) 0.5×. (**a**) Figure showing the two initial sections and the location of the cardiac structure (red circle). (**b**,**c**) Serial sections showing a better view of the two vertebral columns and remaining cardiac tissue in the paraffin block (red circle). Also, note the absence of another cardiac structure on serial sections. Assessment of the internal organs, including the liver and kidneys, was not possible because of advanced autolysis. CP—cephalic pole; vc—vertebral column; r—ribs.

**Figure 4:**
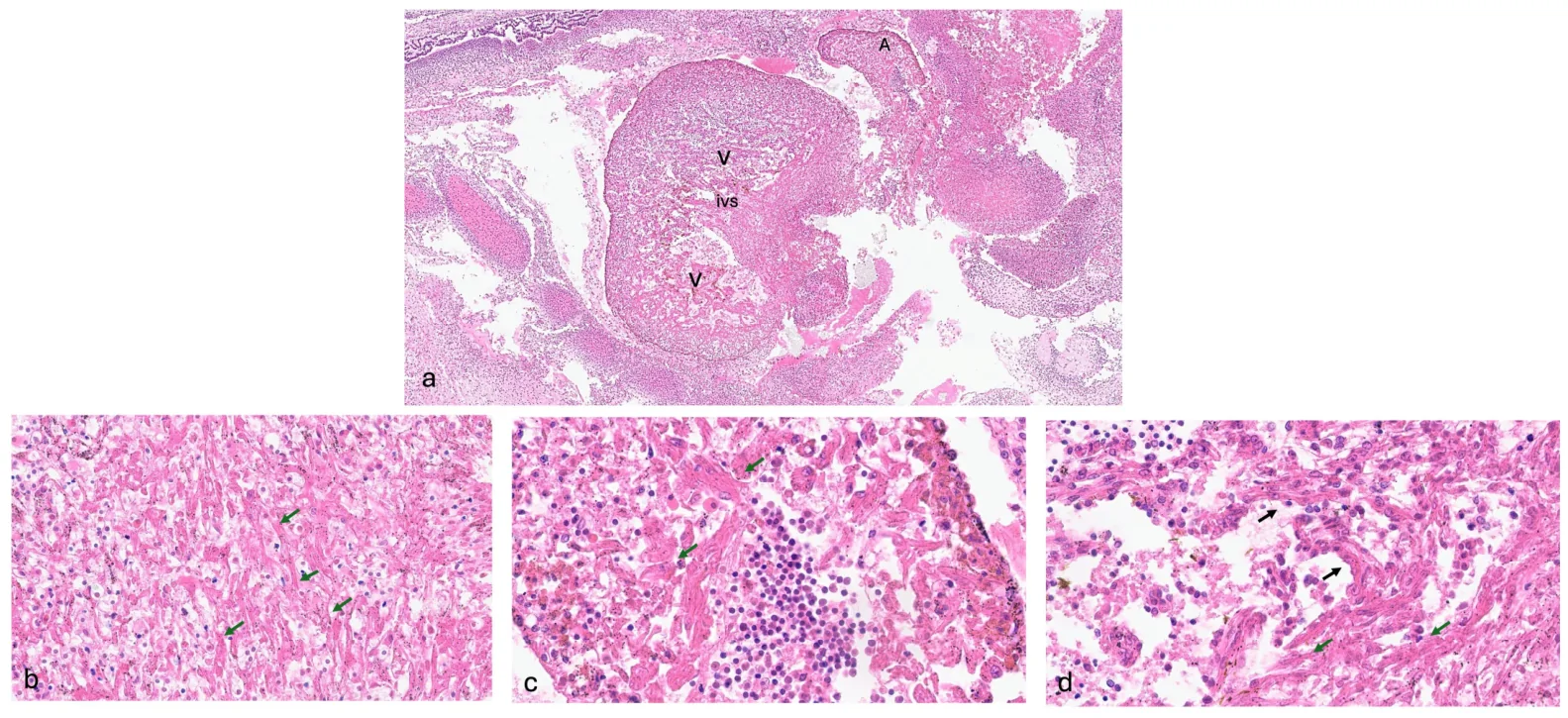
The microscopic postmortem examination revealed in the serial section one multichambered cardiac complex within the common thoracic mass, confirming the prenatal imaging diagnosis. (**a**) Microscopic cardiac examination HE, 40×. Low magnification of the cardiac structure, on a four-chamber view, shows the two ventricles (v) along with the interventricular septum (ivs) and one of the two atria (A). (**b**) HE, 3.5×. High magnification of the ventricular wall, showing the characteristic ramified architecture of the cardiac muscular fibers, with one centrally located nucleus (green arrows). (**c**) HE, 40×. High magnification of the atrium showing cardiac muscle and blood with numerous nucleated erythrocytes and immature blood cells inside the atrial chamber. (**d**) HE, 40×. High magnification of the ventricular chamber wall, showing cardiac muscle (green arrow) and focally endocardium (black arrows). The feasibility of surgical separation of thoracopagus twins depends primarily on the extent of organ sharing, particularly cardiac anatomy. Reported series indicate that the heart is shared in approximately 75% of thoracopagus cases, the pericardium in about 90%, and the liver in nearly 100% [5]. Detailed assessment by fetal echocardiography, postnatal CT angiography, MRI, and three-dimensional reconstruction is essential for surgical planning and multidisciplinary decision-making. Management requires a coordinated multidisciplinary approach involving maternal–fetal medicine specialists, pediatric cardiologists, radiologists, neonatologists, and pediatric surgeons [6]. Prenatal counseling should be structured, evidence-based, and centered on anatomical findings. Parents must be clearly informed that identification of a single shared heart represents a decisive negative prognostic factor, excluding the possibility of long-term survival or surgical separation [7]. Counseling should address prognosis, available management options, and the low recurrence risk associated with the sporadic nature of conjoined twinning. While first-trimester prenatal diagnosis of thoracopagus twins has already been described [8], the present report highlights the added value of combining three-dimensional color Doppler, Silhouette reconstruction, and post-termination histopathological correlation. This multimodal documentation provides a detailed demonstration of shared cardiac anatomy and strengthens confidence in early prenatal diagnosis, offering an educational resource for fetal medicine specialists.

## Data Availability

The original contributions presented in this study are included in the article. Further inquiries can be directed to the corresponding author.

## Supplementary Materials

The following supporting information can be downloaded at https://www.mdpi.com/article/10.3390/diagnostics16162562/s1. Video S1: Transvaginal ultrasound of 8-week and 3 days thoracopagus conjoined twins; Video S2: Cardiac assessment of 8-week and 3 days thoracopagus conjoined twins.
